# Isolation and Pathogenicity of an Emerging Highly Virulent CSFV 2.1c Strain in South China

**DOI:** 10.3390/vetsci12070606

**Published:** 2025-06-21

**Authors:** Xiaopeng Gao, Yu Wu, Yi Song, Feibao Huang, Limiao Lin, Haishen Zhao, Bohua Ren, Qunhui Li, Lang Gong

**Affiliations:** 1 Key Laboratory of Zoonosis Prevention and Control of Guangdong Province, College of Veterinary Medicine, South China Agricultural University, Guangzhou 510000, China; xpgao@stu.scau.edu.cn (X.G.); songyi111@stu.scau.edu.cn (Y.S.); hfb0922@stu.scau.edu.cn (F.H.); 2 Wen’s Food Group, Yunfu 527400, China; wuyu65@mail2.sysu.edu.cn; 3 Yunfu Branch of Guangdong Provincial Laboratory of Modern Agricultural Science and Technology, Lingnan, 527400, China; animsci@126.com (L.L.); 17817887068@126.com (H.Z.); renbaihua2012@126.com (B.R.)

**Keywords:** classical swine fever, genome, pathogenicity, mutation, virulence

## Abstract

Classical swine fever (CSF) is an infectious disease caused by classical swine fever virus (CSFV), which is endemic in many areas of China, causing serious economic losses to pig farms. Currently, 2.1 subgenotype strains are predominantly prevalent in China. Although abundant information is available on 2.1 subgenotype isolates, limited data are available on pathogenicity analysis. In this study, a CSFV strain was isolated from a pig farm in Guangdong Province, China. Whole genome sequencing showed that the strain had a genome length of 12,296 bp, and it was named GD-2024. Based on genetic evolutionary analysis, the strain was categorized into subgenotype 2.1c, and the nucleotide and amino acid homology of the strain with the representative strains of each subgenotype was in the range of 83.1–97.6% and 90.8–99.4%, respectively. Further mutation analysis revealed that the strain had three nucleotide site mutations in the 5′UTR and 3′UTR regions and two amino acid site mutations in the E2 region. The clinical pathogenicity of this strain was investigated. Infection with GD-2024 led to persistent fever and high viremia in pigs as well as inflammatory damage in multivisceral tissues. The mortality rate of infected pigs reached as high as 60%, contradicting the currently reported virulence of 2.1 strains. In summary, we have isolated and reported a subgenotype 2.1c strain with high virulence. Its genomic variation provides a basis for further analysis of virulence determinants and serves as a clinical reference and guide for the prevention and control of CSF.

## Simple Summary

CSFV subgenotype 2.1 strains are prevalent in China, but their pathogenicity is poorly understood. We isolated strain GD-2024 (subgenotype 2.1c) from Guangdong, showing genomic mutations and high virulence in pigs, with persistent fevers, high viremia, organ inflammation, and 60% mortality, contradicting low-virulence reports for subgenotype 2.1. This study highlights a virulent 2.1c strain, providing insights for CSF control and virulence research.

## 1. Introduction

Classical swine fever (CSF) is a highly contagious disease of pigs caused by classical swine fever virus (CSFV). This infectious disease requires notification by the World Organization for Animal Health. CSF outbreaks have caused serious economic losses in the global pig industry [1]. CSFV is a single-stranded positive-stranded RNA virus belonging to the Flaviviridae family of distemper viruses, which also includes bovine viral diarrhea virus and border disease virus [2]. The genome consists of non-coding regions (UTRs) at the 5′ and 3′ ends and a large open reading frame of approximately 12.3 kb [3]. Processing and modification by viral and host cell proteases results in the production of four structural proteins (C, E^rns^, E1, and E2) and eight nonstructural proteins (N^pro^, P7, NS2, NS3, NS4A, NS4B, NS5A, and NSSB) [4]. Swine fever was first reported in 1810 in Tennessee, and the disease rapidly began to spread globally.

At present, it is found in most of Asia, a few countries in Central and South America, and some eastern European countries [5]. CSFV is categorized into three genotypes (1–3) and 11 subgenotypes (1.1–1.4, 2.1–2.3, and 3.1–3.4) [6]. At present, the 2.1 subgenotype is mainly prevalent in mainland China, and this subgenotype has become the dominant prevalent strain across China, including its southern regions [7]. Meanwhile, the 3.4 subgenotype was mainly prevalent in Taiwan prior to 1996, after which this subgenotype was gradually replaced by the 2.1 subgenotype. The 2.1 and 2.2 subgenotypes have also been reported in Vietnam [8]. In conclusion, the long-term endemicity of swine fever is caused by strains of low-to-moderate virulence. China, as a large pig-breeding country, has extensive commercial and mobile pig herds, and some of the breeding pigs are imported from abroad. The high-frequency trade of pigs promotes the spread of CSFV.

Recently, a large number of pigs died in a pig farm in Guangdong Province, and the diseased pigs were positive for swine fever virus. In this study, further isolation and whole-genome sequencing of the positive material were performed to analyze genetic mutations. Simultaneously, the pathogenicity of the strain was studied to establish a complete and detailed pathogenicity model.

## 2. Materials and Methods

### 2.1. Clinical Samples and Virus Isolation

In October 2023, 10 samples from pigs, including spleens, kidneys, and lymph nodes, suspected to be infected with CSFV, were collected on a large swine farm in Guangdong. The clinical features of the pigs included fever, anorexia, and splenic infarction. The virus was isolated using PK-15 porcine kidney cells. Samples from pigs suspected of being infected were ground in Dulbecco’s modified Eagle medium (DMEM [pH 7.2], Gibco, Thermo Fisher Scientific, Waltham, MA, USA), filtered through a 0.22 μm filter, and inoculated in PK-15 cells cultured in DMEM supplemented with 10% fetal bovine serum (FBS; Biochrom, Berlin, Germany) and 1% penicillin/streptomycin (Biochrom). After the infected PK-15 cells were incubated for 3 h, they were supplemented with fresh DMEM containing 2% FBS, followed by continued incubation for 72 h at 37 °C in 5% CO_2_. After three repeated freeze–thaw cycles, the supernatant was collected by centrifugation to remove cellular debris and stored at −80 °C.

### 2.2. Immunofluorescence Assay (IFA)

PK-15 cells were seeded into 96-well plates and infected with GD-2024 [multiplicity of infection (MOI) = 1] for the indicated times. The cells were incubated at 37 °C and 5% CO_2_ for 48 h, followed by two washes with PBS and fixation with pre-chilled absolute ethanol at 4 °C for 30 min. Subsequently, a CSFV-specific monoclonal antibody targeting the E2 protein (Presented by Professor Tu Changchun Research Group of Jilin University) was utilized as the primary antibody and incubated with the cells at 37 °C for 2 h. The cells were then washed and incubated with anti-mouse IgG conjugated with FITC (Sigma-Aldrich, St. Louis, MO, USA) diluted 1:100 with 5% BSA for 1 h at 37 °C. Finally, after four washes with PBST, the cells were observed using a fluorescence microscope (DMi8, Leica, Wetzlar, Germany).

### 2.3. Primer Design and Cloning and Sequencing of Whole-Genome Sequences

The primers (Table 1) were based on the sequences and the distribution of restriction endonuclease sites of CSFV strains (GenBank No. JX262391) and synthesized by Sangon Biotech Co., Ltd. (Shanghai, China). Total RNA was extracted from viral fluids using the Viral RNA mini kit (Qiagen, Hilden, Germany). Reverse transcription (RT)-PCR was performed using the One Step RT-PCR Kit (Qiagen) according to the manufacturer’s instructions. A total of 7 μL of the RNA template was added to 43 μL of the RT-PCR master mix, which included 1 μL each of the primer sets. RT was performed at 52 °C for 30 min, followed by amplification via 40 cycles of denaturation at 98 °C for 10 s, annealing at 49–58 °C for 5 s, and extension at 68 °C for 1 min, and final extension was performed at 72 °C for 10 min. RT-PCR products were analyzed via 1% agarose gel electrophoresis. The target fragments were excised from the gels for purification using a Gel Extraction Kit (Omega Bio-Tek, Norcross, GA, USA) at a later stage. The purified PCR products were cloned into a pMD18-T vector (TaKaRa, Kusatsu, Japan). Recombinant clones were sequenced on an ABI-PRISM Model 377 DNA sequencer (Thermo Fisher Scientific).

### 2.4. Phylogenetic Analysis and Sequence Alignment

A phylogenetic tree with 1000 bootstrap replicates was constructed by the neighbor-joining (NJ) method using Molecular Evolutionary Genetics Analysis software 11.0 (Center for Evolutionary Functional Genomics, The Biodesign Institute, Tempe, AZ, USA). The evolutionary trees of CSFV isolates and other known global isolates were determined on the basis of their full-length E2 gene sequences. The reported nucleotide sequences and deduced amino acid sequences of CSFV were analyzed for homology using DNASTAR software (DNASTAR, Madison, WI, USA). The sequences of all CSFV strains used for comparison and phylogenetic tree analysis, including GD-2024, were obtained from GenBank.

### 2.5. Animal Experiments with the GD-2024 Strain

Ten 35-day-old piglets were purchased from a pig farm in Guangdong Province, and to ensure that all pigs were not infected with CSFV, RT-PCR and an antibody detection kit were used to detect the virus. The pigs were randomly divided into two groups of five pigs each. Pigs in the challenge group were injected intramuscularly with 2 mL × 10^5^ TCID_50_ of the GD-2024 strain, whereas those in the control group were injected intramuscularly with 2 mL of saline. The rectal temperature and clinical signs of experimental piglets were recorded daily to calculate the clinical score (CS). Ten parameters were recorded on a daily basis. Liveliness, body tension, body size, respiration, walking, skin, eyes, appetite, defecation, and fever and were graded according to the following scoring system: 0, normal; 1, slightly altered; 2, showing significant clinical signs; and 3, showing severe CSF signs. Fever was scored as follows: 0, ≤39.5 °C; 1, 39.6–40.0 °C; 2, 40.1–40.9 °C; and 3, ≥41.0 °C. Autopsy was performed on dead or moribund piglets. Skin, subcutaneous tissue, serum, tonsils, spleen, kidneys, lymph nodes, ileum, rectum, and lungs were observed and scored for severity over a range of 0–3. At autopsy, tissues were collected for routine pathological examination and stained with hematoxylin–eosin (HE).

The animal experiments were conducted in accordance with the guidelines of the South China Agricultural University Institutional Animal Care and Use Committee (SCAU-AEC) and were approved by the committee (SCAU-AEC-2023A009).

### 2.6. Histopathology

At necropsy, lung tissues were fixed in 10% buffered neutral formalin for hematoxylin and eosin and immunohistochemical staining, as previously described. Dyeing was performed using a Leica fully automatic dyeing machine (Leica, Wetzlar, Germany). Sample slides were observed under a 200× microscope.

### 2.7. Statistical Analysis

In each experimental group, statistical significance was measured using one-way analysis of variance. Two-sided *p*-values of <0.05 were considered to indicate statistical significance.

## 3. Results

### 3.1. Identification of a New CSFV Strain

**Figure 1:**
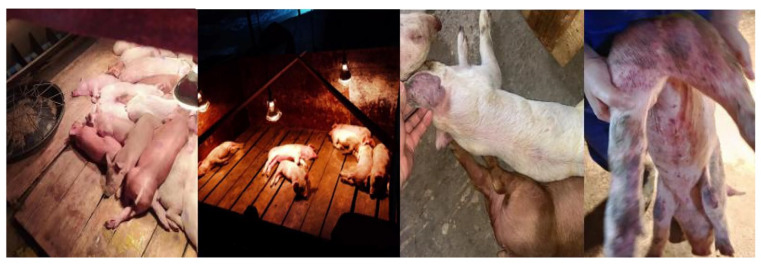
Clinical symptoms of a pig farm in Guangdong.

An acute outbreak of piglet death occurred in a pig farm in Guangdong Province. (Figure 1). Clinical symptoms included red skin, multiple hemorrhages, cyanosis, diarrhea, and loss of appetite. The daily mortality rate significantly increased, reaching up to 90%. Further examination of the diseased pigs showed positivity only for CSFV.

**Figure 2:**
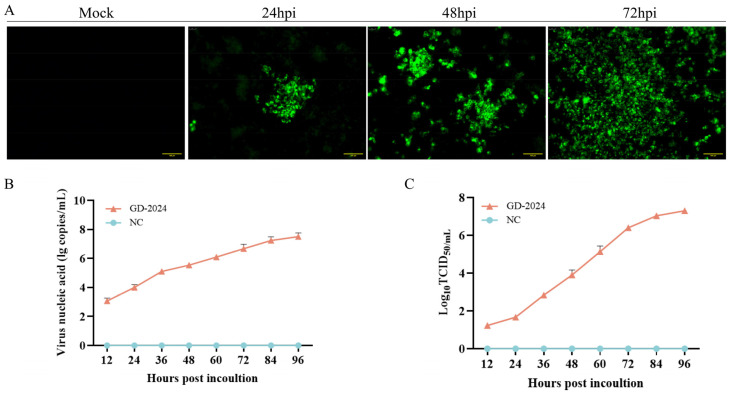
(**A**) IFA revealing the reactivity of a monoclonal antibody against the CSFV E2 protein from GD-2024 at 24, 48, and 72 h post-infection. Scale bars indicate 100 µm. (**B**) GD-2024 viral titer detection at 12, 24, 36, 48, 60, 72, 84, and 96 hpi. (**C**) Growth kinetics of GD-2024.

CSFV-positive samples were diluted with culture medium, passed through 0.22 μm filters, and inoculated onto PK-15 cells for virus isolation. The antigen of strain was identified by indirect immunofluorescence assay using the E2 antibody, resulting in brilliant green fluorescence which was indicative of viral antigen (Figure 2). In summary, a CSFV strain was isolated and named GD-2024.

### 3.2. Analysis of Full-Length Genomic Sequences

**Figure 3:**
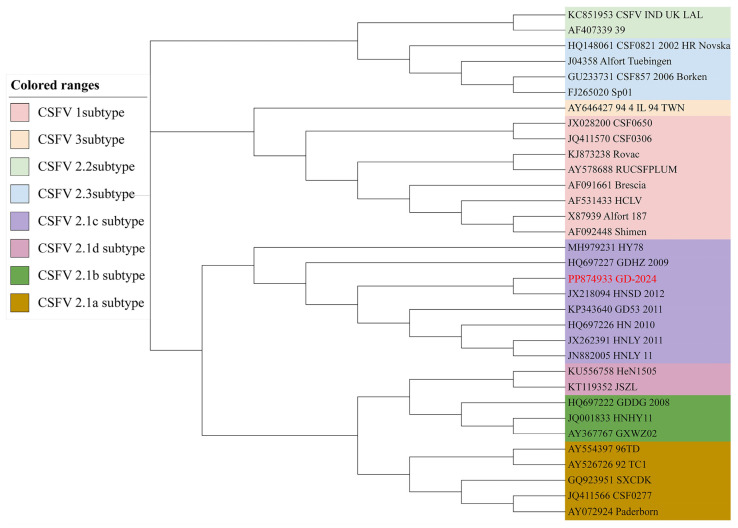
Phylogenetic tree based on the complete E2 sequences of 33 CSFV strains, constructed using the NJ method in MEGA 11.0 software.

The GD-2024 strain was amplified in segments using the six primer pairs listed in Table 1 and sequenced separately, and the full-length spliced genome comprised 12,296 bp. The genomic sequence has been submitted to the NCBI (GenBank accession number PP874933). To reveal the genetic relationship of the new CSFV isolate, the experimentally determined full-length E2 gene sequence of CSFV was phylogenetically analyzed with the E2 gene sequences of domestic and foreign swine fever virus strains of different genotypes or genotypes published in GenBank using MEGA11.0, and the sequences were aligned to create a phylogenetic tree using the NJ method in the software. The maximum composite likelihood was selected as the model, and the bootstrap value was set to 1000 (Figure 3). The phylogenetic tree indicated that the GD-2024 isolate belonged to subgenotype 2.1c. The complete nucleotide sequences of the new isolates were further compared with eight reference CSFV strains, including Shimen (1.1), Paderborn (2.1a), HEBZ (2.1b), HNSD-2012 (2.1c), JSZL (2.1d), CSFV39 (2.2), Alfort/Tuebingen (2.3), and 94.4/IL/94/TWN (3.4), as presented in Table 2. The whole genome of GD-2024 exhibited the highest homology with the subgenotype 2.1c isolate HNSD-2012 (97.6%), whereas its homology with other subgenotype 2.1 isolates ranged from 91.7 to 93%, versus only 83.1% with the subgenotype 3.4 isolate 94.4/IL/94/TWN. A more detailed analysis of genomic features revealed that E2 was the most variable region in the genome, with 82.2–97.9% homology with the other eight representative isolates, and 5′UTR was the most conserved region in the genome, with 89.8–98.1% homology with the other eight representative isolates. At the amino acid level, GD-2024 displayed the highest homology of 98–99.3% with the eight representative isolates in the NS3 region. The detailed homology of the new isolate with the other eight representative isolates is presented in Table 3.

### 3.3. Sequence Analysis of UTRs

**Figure 4:**
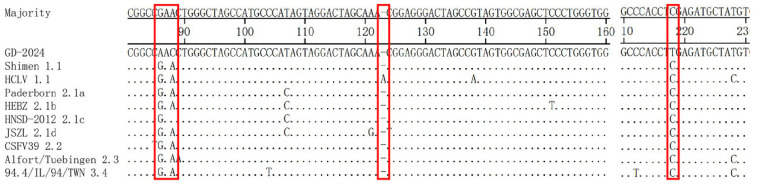
5′UTR sequence comparison of GD-2024 isolate and nine reference isolates. Some mutations or deletion regions of these isolates are indicated by red boxes and are described in detail in the text.

The 5′UTR is the most conserved region in the CSFV genome, and the 5′UTR of GD-2024 had mutations at positions 86 (G to A), 88 (A to C), and 218 (C to T) compared with the other subgenotypes (Figure 4). The vaccine strain HCLV had a 1-nucleotide (A) insertion at nucleotides 122–123 compared with the other subgenotypes.

**Figure 5:**
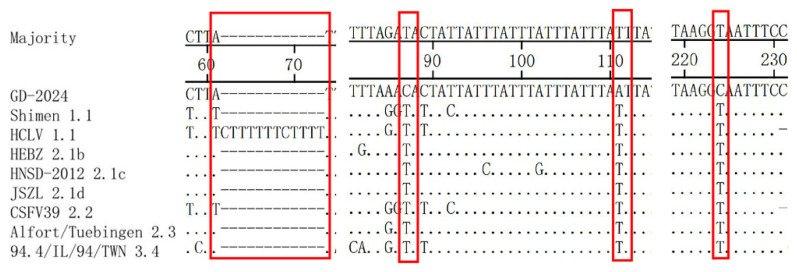
3′UTR sequence comparison of GD-2024 isolate and eight reference isolates. Some mutations or deletion regions of these isolates are indicated by red boxes (□) and are described in detail in the text.

Sequence comparisons illustrated that the 3′UTR of GD-2024 had mutations at positions 87 (T to C), 111 (T to A), and 224 (T to C) compared with the other subgenotypes (Figure 5). The vaccine strain HCLV contained a 12-nucleotide (CTTTTTTTCTTTT) insertion at nucleotides 61–62 compared with the other subgenotypes.

### 3.4. Amino Acid Analysis of E2

**Figure 6:**
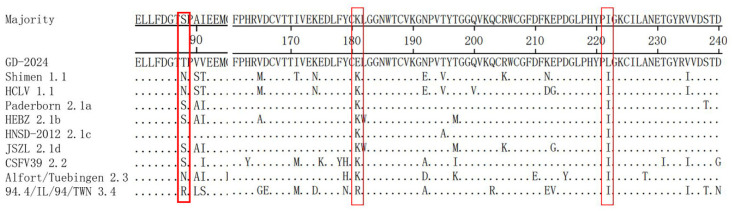
Amino acid sequence comparison of the E2 gene between the GD-2024 isolate and nine reference isolates. Some mutations or deletion regions of these isolates are indicated by red boxes (□) and are described in detail in the text.

The E2 amino acid sequences of GD-2024 and nine reference isolates were comparatively analyzed and the results (Figure 6) indicated that the new isolate had variations at positions 181 (K/R to E) and 222 (T to L). Additionally, the monoclonal antibody determinant cluster on the surface of the E2 protein could be divided into four antigenic structural domains named A, B, C, and D. Among these, the B/C antigenic structural domains were located at amino acids 1–90, and the D/A antigenic structural domains were located at amino acids 91–170. Sequence comparisons revealed the absence of complete substitutions in the antigenic structural domains of E2 in the GD-2024 strain.

### 3.5. Pathogenicity Analysis of GD-2024

**Figure 7:**
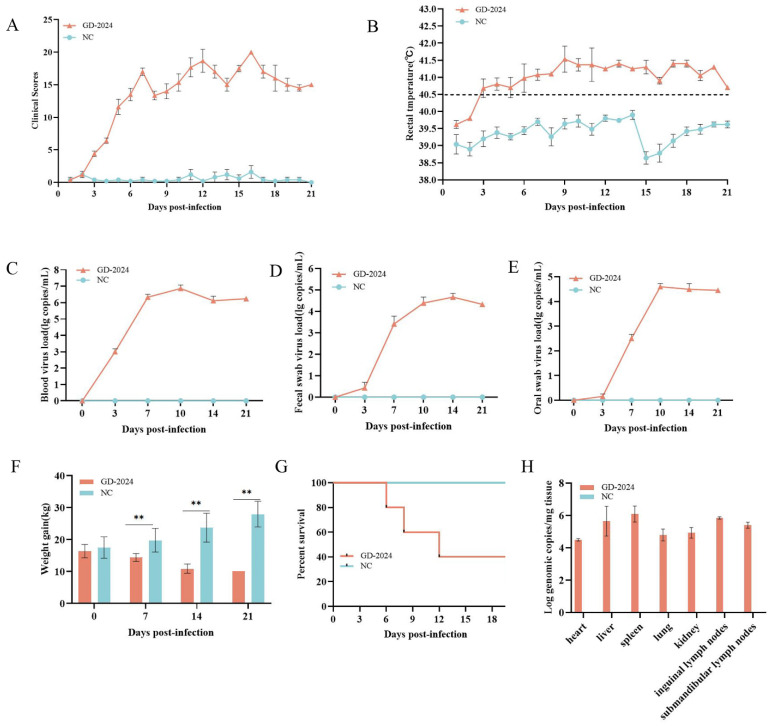
(**A**) Clinical scores of piglets after challenge throughout the experiment. (**B**) Body temperature changes in piglets in each group after challenge. (**C**–**E**) Viral load detection in the blood, oral swabs, and fecal swabs. (**F**) Changes in body weight of piglets in each group after challenge. (**G**) Survival rate of pigs in each group during the challenge study. Each bar represents the mean ± standard deviation in each group. (**H**) Quantification of CSFV genomic RNA in the CSFV GD-2024 strain-challenged pigs. Data are presented as mean ± standard error (n = 5 per group). Two-way ANOVA was used for analysis. Significant differences are marked with asterisks: ** *p* < 0.01 compared with the negative control group.

Piglets in the challenge group exhibited typical clinical symptoms of swine fever virus infection, including anorexia, depression, chills, movement disorders, diarrhea, and high fever. Such symptoms were not observed in the control group. The CS of pigs infected with the GD-2024 strain was 15–25, indicating high virulence (Figure 7A). In addition, on the third day after viral challenge, the piglets displayed obvious fever symptoms, with a rectal temperature exceeding 40.5 °C and peaking at 9 days post-infection (dpi) (Figure 7B). Conversely, the piglets in the control group maintained a normal body temperature throughout the observation period, with a rectal temperature of 38.5–39.9 °C. Oral swabs, fecal swabs, and anterior vena cava blood samples were collected on days 0, 3, 7, 10, 14, and 21 dpi for detoxification monitoring and viremia monitoring. Results indicated rapid viremia increase at 3 dpi, peaking at 10 dpi. At the same time, viral nucleic acid was detected in both oral and anal swabs, indicating that GD-2024 can cause high levels of viremia and shed orally and in feces (Figure 7C–E). Body weight gradually decreased throughout the observation period in the challenge group, whereas the weight of piglets in the control group increased normally (Figure 7F). During the experimental period, one piglet each died at 6, 8, 12, and 20 dpi in the challenge group, but no piglets died in the control group. The survival rate of piglets was 40% in the GD-2024 group, compared with 100% in the blank control group (Figure 7G).

**Figure 8:**
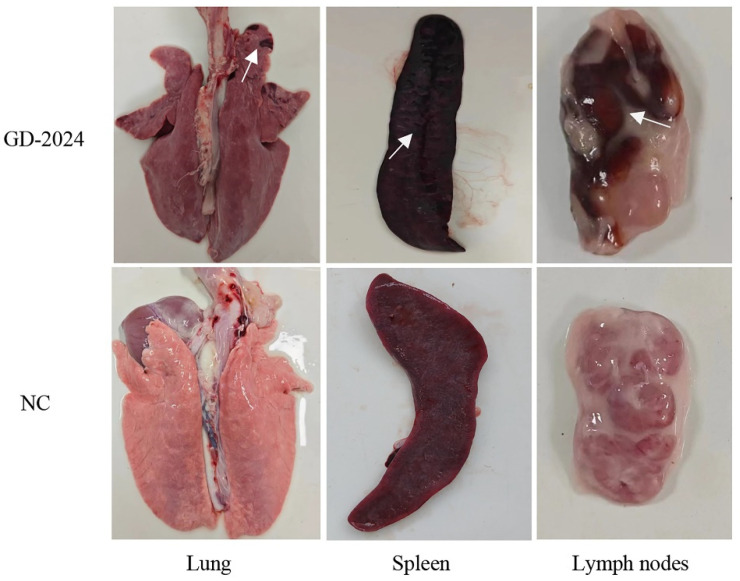
Representative gross lesions in the pigs inoculated with the CSFV GD-2024 strain. The ocular lesions observed in the GD-2024 challenge group exhibited solid lesions in the lungs, infarcts in the spleen, and hemorrhages in the lymph nodes. In contrast, the control group did not exhibit any lesions. The white arrow indicates the location of the tissue lesion.

**Figure 9:**
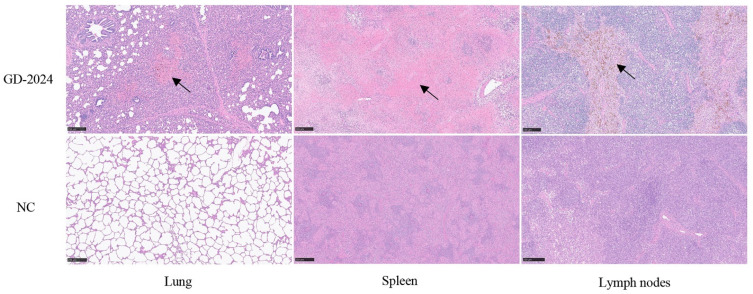
Histopathologic lesion results in organs of pigs infected with the CSFV GD-2024 strain. The GD-2024 challenge group exhibited interstitial pneumonia, splenic erythrocyte leaching, and lymphocyte necrosis, whereas the control group did not display any lesions. The black arrow indicates the location of the lesion. Scale bars indicate 250 µm.

Post-experiment, euthanized pigs were examined for organ viral load, with spleens showing the highest viral load (≥10^6^ copies/mg), followed by lymphoid tissues and livers (10^4^–10^6^ copies/mg) (Figure 7H). Ocular lesion results indicated severe solid lesions in the lungs of mice in the challenge group (Figure 8), with the whole lung being dark red and the lobules exhibiting reddish-brown solid lesions. Foci of infarctions of varying sizes were observed on the surface and margins of the spleen, and the whole spleen was dark black. Additionally, the lymph nodes were severely hemorrhagic and enlarged. There were no lesions in the control group. Further HE staining and pathological section analysis indicated that the alveolar cell spaces disappeared, and a large amount of inflammatory infiltration was present (Figure 9). The spleen was obviously hemorrhagic, the medulla was blurred, the lymphocytes were obviously reduced in number with an increased density, and a small number of nuclear schizophrenic images were observed. The lymph nodes were vasodilated with erythrocyte leakage, the lymphocytes were necrotic and collapsed, lymphocyte counts in the germinal center were reduced, and a small number of nuclear schizophrenic images were observed. The test results illustrated that the GD-2024 strain could cause severe tissue damage and lesions in pigs.

## 4. Discussion

The CSFV 2.1 subgenotype was first reported in China in 1979 [9], and a large-scale outbreak of this subgenotype occurred in Europe in the late 1990s, which later evolved into the 2.1a subgenotype [10]. During the same period, the subgenotype of CSFV epidemic strains in Taiwan shifted from 3.4 to 2.1, and in 2005, scholars in Taiwan classified swine fever virus strains isolated from 1993 to 2001 into the 2.1a and 2.1b subgenotypes. In addition, in South Korea the prevalent strains of CSFV shifted from the 3.2 subgenotype to the 2.1b subgenotype [11]. In 2008, Chen et al. reported that 34 of 35 prevalent strains of CSFV from southeastern China belonged to the 2.1b subgenotype [12,13]. In 2013, Jiang et al. classified five strains isolated in Hunan Province based on the full-length E2 sequence and a partial fragment of the E2 gene (216 nucleotides) into a new subgenotype, namely 2.1b, and found that the 2.1c subgenotype has been prevalent in Hunan and Guangdong since 2011 [14]. In 2014, Zhang et al. reported that 15 prevalent CSFV strains from Shandong belonged to a new subgenotype named 2.1d, which was genetically established through sequencing of the full E2 sequence, partial E2 sequence, and partial NS5B sequence [15]. Gong et al. analyzed the genetic diversity of 39 isolates from Guangdong and Guangxi and isolates from other regions of China and abroad from 2004 to 2012 and further classified the 2.1 subgenotype of swine fever strains into 10 subgenotypes by adding 2.1e, 2.1f, 2.1g, and 2.1f to the previously reported 2.1e, 2.1f, 2.1g, 2.1h, 2.1i, and 2.1j subgenotypes [16]. The two Hunan isolates HNZH2011 and HNHY11, which were classified as 2.1b by Jiang et al., were reclassified as 2.1j and 2.1h, respectively. The five Guangdong isolates, which were classified as 2.1b by Peng et al., were reclassified as 2.1i, and the six isolates that were classified as 2.1d were reclassified as 2.1d. Six isolates classified into 2.1d were reclassified as 2.1g. In recent years, the genotypes of CSFV strains have been changing, and multiple genotypes have been circulating simultaneously, which makes the detection and control of swine fever increasingly complex. In this study, a strain named GD-2024 with a genome of 12,296 bp was successfully isolated from clinical samples, and further genetic evolutionary analyses revealed that it belonged to the subgenotype 2.1c.

The 5′UTR of CSFV is 373 bp in length. It is mainly responsible for regulating the replication and translation of viral RNA, and it contains an IRES at nucleotides 60–375 that binds to the ribosome to initiate protein translation [17,18]. It was found that the IRES contains binding sites for NS3 and NS5A, and these two nonstructural proteins might be involved in both viral replication and translation [19]. In addition, the regulation of viral replication and translation by an IRES has been reported in hepatitis C virus, which belongs to the same Flaviviridae family as CSFV [20]. In the present study, we found that the new isolate carried the mutations 86A, 88C, and 218T in the 5′UTR, which interestingly were all located in the IRES, compared with other representative strains.

The 3′UTR of CSFV is 228 bp in length and rich in uracil [21]. It is extremely important for viral reproduction and is involved in the replication of viral RNA, the transcription and translation of polyproteins, and the assembly of mature viral particles. Research confirmed that the poly-T insertion in the 3′UTR is crucial for attenuating CSFV virulence [22]. The 3′UTR of HCLV (C strain), a weak rabbit vaccine strain of swine fever, featured 13 nucleotide insertions, which were found to be related to the gradual adaptation of the virus to the rabbit body [21,23], whereas Li et al. reported that the non-coding region of the C strain was indispensable for causing fever in rabbits, although it was not essential for viral replication and adaptation to the rabbit body [24]. In this study, we found that the new isolate lacked the poly-T insertion. Moreover, the GD-2024 strain isolated in the present study carried the 87C, 111A, and 224C mutations in the 3′UTR, which could potentially impact the proliferation ability of the strain.

As the main protective antigen of CSFV, E2 is involved in the process of viral infection, and it carries antigenic determinants that can stimulate the production of CSFV-neutralizing antibodies [25,26]. Meanwhile, E2 determines the difference in virulence between different strains of CSFV [27]. As early as 1989, Wensvoort et al. used multiple CSFV-specific monoclonal antibodies to classify the antigenic domains of the E2 protein into four separate regions: A, B, C, and D [28]. Later, Van Rijn et al. used model predictions to determine that these antigenic domains are located in two relatively independent antigenic units, one consisting of the structural domain BC and the other consisting of highly conserved A [29]. The linear B-cell epitopes at amino acids 64–70 and 83–89 are located in the antigenic structural domains B and C, respectively. Multiple sequence comparison analyses indicated that the B-cell epitope at amino acids 64–70 was highly conserved across strains, with amino acid substitutions only present in a few individual strains. Peng et al. investigated the response of this B-cell epitope to a monoclonal antibody, finding that after shifting the amino acid at position 88 from N to S, the reactivity of the monoclonal antibody with this epitope was significantly reduced [30]. In this study, the new isolate had an N-to-T substitution at amino acid position 88, which is consistent with the amino acid substitution results of the 2.1c subgenotype strain in the study of Jiang et al. This mutation might affect the virulence of the strain. Additionally, the linear epitope TAVSPTTLR located at amino acids 829–837 of the E2 protein is a highly conserved specific epitope in CSFV [31]. Mutation of this epitope can lead to mild clinical symptoms in infected pigs, highlighting its role as a key virulence factor [32]. Importantly, the epitope in the E2 protein of the new isolate showed no changes.

Next, the pathogenicity of the newly isolated CSFV strain was evaluated in this study using the virulence assessment model developed by Mittelholzer et al. [33]. The findings included significant febrile symptoms, a high mortality rate (80%), and significant lesions in various tissues and organs on the third day after viral challenge, and the results supported the idea that the GD-2024 strain was a highly virulent CSFV strain (15 < CS < 25). However, several previous studies have reported that CSFV genotype 2 is less pathogenic than genotype 1, a highly virulent genotype, and in the study by Hao et al., the 2.1c and 2.1d subgenotypes were moderately pathogenic [34]. However, there does not appear to be a necessary link between genotype and virulence, and strains that are genetically similar might also exhibit significant differences in virulence. In the study by Gong et al., five field isolates belonging to genotype 2 consisted of three highly virulent and two moderately virulent strains. Although the JL23 and HuB16 strains were genetically categorized into subgenotype 2.1b, the JL23 isolate (CS = 25.2) was deemed more virulent than the Shimen strain (CS = 16.8) [35]. This study investigated the biological characteristics and clinical pathogenicity of newly emerging mutant strains of classical swine fever virus. Virulence variations observed among strains within the same evolutionary branch may be attributed to key nucleotide mutations that enhance viral pathogenicity. In subsequent work, reverse genetics techniques will be employed to validate this hypothesis and elucidate the precise relationship between strain-specific mutations and virulence.

In recent years, there have been limited reports of CSFV isolates in the field. In this study, a CSFV strain was isolated from infected pigs, and genetic evolutionary analysis revealed that the strain was genetically classified into subgenotype 2.1c, featuring genomic variants in its UTRs and E2 protein. The strain was highly pathogenic in piglets, causing obvious fever, lesions in multiple organs, and leading to death in 60% of pigs. Further genetic and pathogenicity analyses of CSFV strains will help us to understand their characteristics, improve prevention and control strategies, and provide guidance for pig production.

## Figures and Tables

**Table 1:** Nucleotide sequences of primers used for amplification of isolate CSFV-GD-2024.

Primer	Primer Sequence (5′-3′)	Position in Genome	Product Size (bp)
F1 and R1	ATACGAGGTTAGTTCGTTCTC CCCTATCTTCCTTGTCACATT	1–2059	2059
F2 and R2	CCCCAGAAACGGCTAGTATG AGTTGTAAACCGGCAGCAAG	2022–3670	1639
F3 and R3	AACAACCCAGTTAAGACCAT GTACCTCTTGGCATAACACC	3640–5284	1645
F4 and R4	CCTAAAGATAAGAAGAGGGTTG ATTCGGAGCGTACTGTAAAT	5256–7496	2241
F5 and R5	ATACTGTGGCAGACTATGTGA ATTTCAGCAGAGCGGTGTCG	7464–9652	2190
F6 and R6	CAGAGGCACGATGGTTGTTG GGCCGTTAGGAAATTACCTTAGTC	9619–12,296	2678

**Table 2:** Gene homology analysis of GD-2024 and other representative CSFV isolates (%).

Nucleotides	Shimen (1.1)	Paderborn (2.1a)	HEBZ (2.1b)	HNSD-2012 (2.1c)	JSZL (2.1d)	CSFV39 (2.2)	Alfort/Tuebingen (2.3)	94.4/IL/94/TWN (3.4)
5′UTR	91.4	96.2	94.1	98.1	95.2	91.7	93.5	89.8
N^pro^	85.9	91.3	90.3	97.4	91.9	86.5	87.3	83.5
C	82.8	92.9	89.9	97.3	90.9	87.2	87.9	83.2
E^rns^	84.3	95.2	93.5	97.2	93.4	88.7	89.4	82.4
E1	82.6	92	90.8	98.1	91.3	88.2	88	81
E2	82.7	91.8	89.5	97.9	89.3	86.1	86.2	82.2
P7	80.5	94.3	93.8	99.5	91.9	91	91	85.7
NS2	83.4	92.4	91.7	97.5	91.4	88.2	88.1	80.5
NS3	87.4	93.7	92.5	98	92.8	90.5	90.9	85.6
NS4A	85.4	92.2	92.2	97.4	92.2	86.5	85.4	82.3
NS4B	86.6	92.4	90.6	97.2	90.3	89.8	89.4	83.8
NS5A	83	92.4	91.2	97.3	90.4	82.9	88.7	80.8
NS5B	84.1	93.8	92.9	97.6	92.5	84.4	88.8	83.7
3′UTR	85	93.1	91.2	96.5	93.8	84.5	91.2	83.6
Complete	84.7	93	91.8	97.6	91.7	87.2	89	83.1

**Table 3:** Amino acid homology analysis of GD-2024 and other representative CSFV isolates (%).

AA	Shimen (1.1)	Paderborn (2.1a)	HEBZ (2.1b)	HNSD-2012 (2.1c)	JSZL (2.1d)	CSFV39 (2.2)	Alfort/Tuebingen (2.3)	94.4/IL/94/TWN (3.4)
N^pro^	91.7	95.8	93.5	98.8	95.8	92.3	89.9	90.5
C	89.9	92.9	91.9	99	91.9	89.9	90.9	84.8
E^rns^	90.3	97.4	96.9	98.2	97.4	95.6	95.6	91.6
E1	93.8	96.4	95.9	99	95.4	95.4	96.4	90.8
E2	90.3	97.6	95.2	99.5	95.7	92	92.5	90.3
P7	90	98.6	95.7	99	94.3	95.7	95.7	94.3
NS2	89.3	96.7	96.3	98.9	94.5	95.2	93.9	88.2
NS3	98	99	98.4	99.3	99	98.4	98.7	98.4
NS4A	95.3	95.3	95.3	98.4	96.9	96.9	96.9	95.3
NS4B	96.3	98.8	98.6	99.4	98.8	97.1	98.6	94.8
NS5A	87.9	95	94.2	98.4	93.6	87.9	92.4	86.5
NS5B	92.6	98.5	96.9	99.4	97.4	93.3	96.5	90.8

## Data Availability

The datasets presented in this study can be found in online repositories. The names of the repository/repositories and accession number(s) can be found at https://www.ncbi.nlm.nih.gov/PP874933, accessed on 10 May 2025).
